# Draft Genome Sequence of Three Endophyte Strains of *Pseudomonas fluorescens* Isolated from *Miscanthus giganteus*

**DOI:** 10.1128/genomeA.00965-16

**Published:** 2016-10-13

**Authors:** António S. Moreira, Kieran J. Germaine, Andrew Lloyd, Richard D. Lally, Paul T. Galbally, David Ryan, David N. Dowling

**Affiliations:** a EnviroCORE, Institute of Technology Carlow, Carlow, Ireland; b TEAGASC Crop Research Institute, Oak Park, Carlow, Ireland

## Abstract

We report here the draft genome sequence of three *Pseudomonas fluorescens* strains (L111, L228, and L321) isolated from *Miscanthus giganteus*. The draft genome analyses uncovered a group of genes involved in the biosynthesis of secondary metabolites and for plant growth promotion.

## GENOME ANNOUNCEMENT

The use of microbial inoculants by the agricultural sector is increasing, as their use has the potential to reduce chemical input costs, help compliance with environmental legislation, and potentially lead to an increase in forage/grain yield (1, 2). Beneficial microbes promote plant growth and/or suppress plant diseases through a variety of mechanisms, which include improved nutrient acquisition, production of growth regulators, and biosynthesis of pathogen-inhibiting compounds (3). Strains of *Pseudomonas* spp. and *P. fluorescens* have been found particularly effective in increasing root and shoot elongation in oilseed rape (*Brassica napus*) (4) and in boosting grain yields in rice and wheat (5).

We have isolated, identified, and thoroughly characterized a number of potential biofertilizer strains from *Miscanthus giganteus*, which showed promising plant growth–promotion results in glasshouse trials. From these trials, we have selected three strains of *Pseudomonas* spp. that exhibited the greatest plant growth–promotion activity, namely, strains L111, L228, and L321. To investigate key plant growth–promotion mechanisms and core genes involved in colonization and biosafety issues, the genomes of these three strains were sequenced. The strains were grown in nutrient broth medium for 18 h, and DNA extraction was conducted according to the manufacturer’s protocol for the Wizard genomic purification kit (Promega). The genomes were sequenced by the Centre for Genomic Research (University of Liverpool, United Kingdom) using the Illumina HiSeq2000 system with a paired-end library. The adapter-trimmed files were quality-trimmed using Sickle (https://github.com/najoshi/sickle), and assembled *de novo* using SPAdes version 2.4 (6). Reads greater than 500 bp were used to assemble the genomes.

The draft genome sequence of L111 consists of 6.72 Mb in 177 contigs (*N*_50_ = 73,901 bp). L228 consists of 6.28 Mb and a 77,900-bp plasmid in 63 contigs (*N*_50_ = 23,2217 bp), and L321 consists of 6.75 Mb in 162 contigs (*N*_50_ = 81,368 bp). These accounted for 99.3%, 99.6%, and 99.3% of the hypothesized genome contents of L111, L228, and L321, respectively. All three draft genomes have a 60.8% GC content, similar to other *Pseudomonas* genome (7). A pseudochromosome for each strain was generated by ordering the contigs based on alignment against the genome of *P. fluorescens* strain SBW25 (8) using BLAST (9). The Bacterial Annotation System (BASys) (10) server version was used to predict and annotate the genes on the draft genomes. A preliminary analysis of the three genomes revealed the presence of important coding genes involved in plant–microbe interactions. These include the secretion system type III, which is believed to be involved in plant–bacterial interactions, the 1-aminocyclopropane-1-carboxylate (ACC) deaminase, which may suggest contributions to plant development under stress conditions, and the plant phytohormone IAA, which is involved in plant stem and root growth regulation.

### Accession number(s).

The draft genome sequence of *Pseudomonas fluorescens* strains L321, L111, and L228, as well as the L228 plasmid, has been deposited in GenBank under the accession numbers CP015637, CP015638, CP015639, and CP015640, respectively.
